# Prospects of Germline Nuclear Transfer in Women With Diminished Ovarian Reserve

**DOI:** 10.3389/fendo.2021.635370

**Published:** 2021-02-22

**Authors:** Antonia Christodoulaki, Annekatrien Boel, Maoxing Tang, Chloë De Roo, Dominic Stoop, Björn Heindryckx

**Affiliations:** ^1^ Ghent-Fertility and Stem cell Team (G-FaST), Department for Reproductive Medicine, Ghent University Hospital, Ghent, Belgium; ^2^ Reproductive Medicine Center, The First Affiliated Hospital of Xiamen University, Xiamen, China

**Keywords:** diminished ovarian reserve, poor ovarian response, oocyte quality, germline nuclear transfer, spindle transfer, polar body transfer

## Abstract

Diminished ovarian reserve (DOR) is associated with a reduced quantity and quality of the retrieved oocytes, usually leading to poor reproductive outcomes which remain a great challenge for assisted reproduction technology (ART). Women with DOR often have to seek for oocyte donation, precluding genetically related offspring. Germline nuclear transfer (NT) is a novel technology in ART that involves the transfer of the nuclear genome from an affected oocyte/zygote of the patient to the cytoplast of an enucleated donor oocyte/zygote. Therefore, it offers opportunities for the generation of genetically related embryos. Currently, although NT is clinically applied only in women with serious mitochondrial DNA disorders, this technology has also been proposed to overcome certain forms of female infertility, such as advanced maternal age and embryo developmental arrest. In this review, we are proposing the NT technology as a future treatment option for DOR patients. Strikingly, the application of different NT strategies will result in an increase of the total number of available reconstituted embryos for DOR patients.

## Introduction

Ovarian reserve refers to the number of primordial follicles residing in the ovary and determines the reproductive lifespan of a woman (1). The number of follicles is predetermined at birth, with female ovaries containing approximately 1,000,000 immature oocytes (2). After birth, this number progressively decreases due to apoptosis and cyclic recruitment of follicles in every menstrual cycle, until menopause, after which the number of follicles will have been reduced to about 1,000 (3). Reproductive efficiency decreases over the years due to this follicular loss, but also as a result of the decreasing quality of the remaining oocytes due to reported increased aneuploidy rates, low fertilization competence, compromised mitochondrial function and higher spontaneous abortion risk (1, 4, 5). The efficiency of the female reproductive outcome reaches a peak in the mid-20s and starts declining slowly, dropping dramatically after 37 years of age (4, 6).

Diminished ovarian reserve (DOR) is characterized by a decrease in the quantity of the ovarian follicular reserve and is mostly attributed to the advanced age of the patient, but also to non-physiological parameters, such as genetic background, surgical interventions, therapies for cancer treatment (7–13). Increased levels of Follicle stimulating hormone (FSH >10 mIU/ml), low Antral follicle counts (AFC <5–7 follicles), and decreased Anti-Mullerian hormone (AMH) levels (<0.5–1.1 ng/ml) are markers for DOR diagnosis (14). Patients with DOR have an occurrence of 31% in ART (15) cycles and are considered as challenging as they usually display poor ovarian response (POR), leading to a lower number of retrieved oocytes and subsequently fewer embryos available for transfer, with resulting poor pregnancy and life birth rates (LBR) (16). Bologna and POSEIDON (Patient-Oriented Strategies Encompassing IndividualizeD Oocyte Number) criteria are being used as means to identify and treat these patients (17–19).

The incidence of POR patients in the ART setting might vary between 6 and 35% (20) while it can be over 50% in women over their forties (21). Several treatment strategies for these patients are being investigated as means to increase the oocyte yield and improve the LBR. These approaches include changes in the type and dose of gonadotropins, stimulation protocols, the use of adjuvants, double stimulation cycles and the manipulation of ovarian tissue for primordial follicle activation. The choice of the appropriate treatment depends on the characteristics of the patient (22–30). Despite the current attempts to improve the poor ovarian response to hormonal stimulation (18, 31, 32), current strategies remain experimental or inconclusive. Child adoption or oocyte donation remains the only options for some patients to fulfil a child wish (33, 34).

DOR in aged women has also been associated with concomitant poor oocyte quality (35–37). Poor oocyte quality is linked to cytoplasmic insufficiency, as several cytoplasmic factors, including mitochondria, metabolites, maternal RNAs and proteins, are important regulators of oocyte and embryo competence (38). In comparison with the quantitative decrease in oocyte numbers, oocyte quality is not easy to assess. In the ART setting, oocyte quality has been linked to morphological features of the oocytes, embryo arrest, blastulation, implantation, pregnancy, miscarriage, and euploidy rates. The ultimate marker is the live birth of a healthy offspring (36). Oocyte quality does not seem to be affected in young DOR patients, when compared to age matched groups with a normal ovarian reserve (37, 39, 40). On the contrary, DOR patients of advanced maternal age have significantly lower chances for a clinical pregnancy when compared to younger DOR patients, higher miscarriage rates and lower high-quality embryos, suggesting poor oocyte quality (40).

Current approaches for assisting patients with DOR and POR could be expanded with the novel germline nuclear transfer (NT) technology. NT offers the possibility to transfer the genetic material of a patient’s oocyte/zygote with compromised cytoplasm to the cytoplasm of an enucleated oocyte/zygote of a healthy donor. This technology enables the generation of embryos, to which both parents have contributed genetically (41). NT is currently clinically applied in a strict subset of patients suffering from mitochondrial DNA (mtDNA) disorders, aiming to overcome maternal mutant mtDNA transmission to the next generation. Besides, increasing interest has been shown in the application of NT to overcome certain types of female-related infertility (42), which is supported by some promising studies in animal models (42, 43), but still, peer-reviewed reports in human patients remain scarce. The rationale of this treatment is that by transferring the nuclear DNA from an oocyte with inferior quality cytoplasm to an oocyte with more competent cytoplasm, like the ones of young, fertile women, could potentially improve embryo development. In this review, we propose the NT technology as a possible novel treatment method for DOR patients in order to increase the number or/and quality of the retrieved embryos.

## Different Nuclear Transfer Techniques

For normal fertilization to occur, an oocyte needs to be at the correct maturation state, both cytoplasmic as nuclear. Before ovulation, oocytes are arrested in meiotic prophase I. They are diploid, having both paternal and maternal genomes, and DNA resides in the nucleus (germinal vesicle (GV)). Following meiotic maturation, oocytes arrest in the metaphase of meiosis II (MII oocyte). Chromosomes are aligned on the spindle, while the oocyte extrudes the first Polar body (PB1) containing the homologous chromosomes. At this stage, the oocyte can be fertilized by the sperm. Following fertilization, the oocyte completes Meiosis II, extruding first a second Polar body (PB2) and forming the maternal and paternal pronuclei, generating the zygote. The PB2 contains the haploid sister chromatids of the maternal pronucleus (44). The NT technology can be performed at different stages of oocyte maturation: Germinal vesicle transfer (GVT), Spindle transfer (ST) or Polar body 1 transfer (PB1T) or at the zygote stage: Pronuclear transfer (PNT) or Polar body 2 transfer (PB2T).

**Figure 1:**
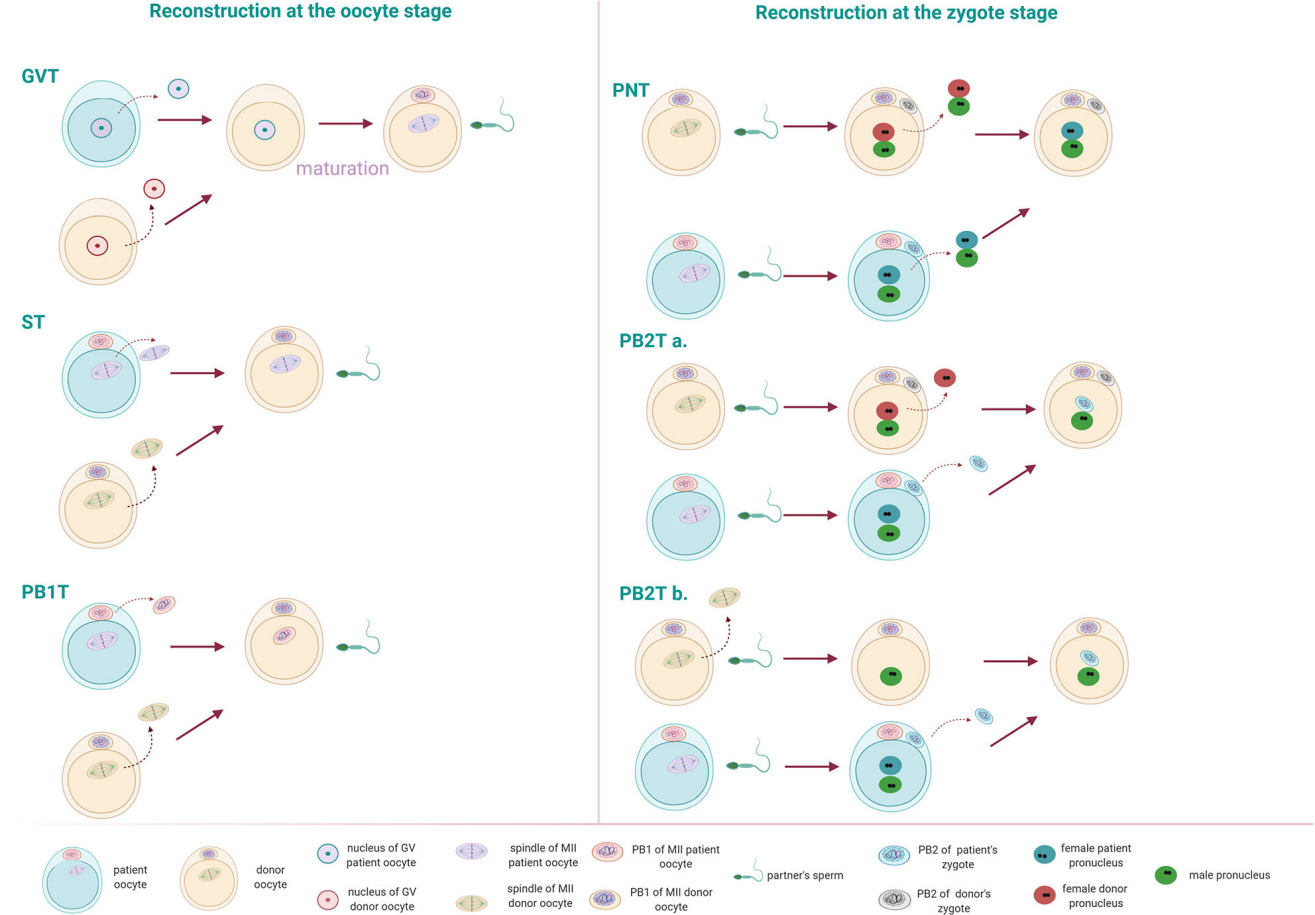
Different nuclear transfer (NT) techniques can occur at the oocyte or the zygote stage. Reconstruction at the oocyte stage: Germinal vesicle transfer (GVT): the nucleus is transferred in an enucleated GV oocyte. Following *in vitro* maturation, the reconstructed oocyte can be fertilized by the patient’s partner sperm. Spindle transfer (ST): The spindle from a mature oocyte (MII) is transferred to the enucleated MII donor oocyte. Polar body 1 transfer (PB1T): The first polar body from an MII oocyte is transferred to an enucleated MII oocyte of a donor. Reconstruction at the zygote stage: Pronuclear transfer (PNT): the pronuclei from a fertilized oocyte are transferred to the enucleated donor zygote. Polar body 2 transfer (PB2T): The second polar body of a fertilized oocyte is transferred to a zygote containing the male pronucleus of the patient’s partner. The paternal pronucleus can occur in two ways: (a) by fertilization of a donor’s oocyte. Following fertilization, the male and female pronuclei form. The female pronucleus of the donor can be removed and replaced by the second polar body of the patient’s MII oocyte. (b) The donor’s MII oocyte is enucleated and injected with the partner’s sperm. Following the formation of the male pronucleus, the second polar body from a patient’s zygote can be transferred.

During GVT, the nucleus of a GV oocyte is being transferred in an enucleated GV donor oocyte (**Figure 1**). The reconstructed oocyte needs to undergo *in vitro* maturation before it can be fertilized by the sperm (45). In ST, the spindle-chromosome complex (containing the DNA) of an MII oocyte is transferred to an enucleated (DNA free) MII donor oocyte which is subsequently fertilized by the sperm of the patient’s partner (46) (**Figure 1**). At the MII stage, also PB1T may occur. PB1T includes the transfer of the first polar body into an enucleated MII oocyte, followed by fertilization (**Figure 1**). In PNT, the pronuclei from the patient’s zygote can be transferred in the cytoplasm of an enucleated donor zygote that will serve as a recipient (46) (**Figure 1**). At this stage, also PB2T may occur, which involves the transfer of PB2 in an enucleated donor oocyte containing only the male pronucleus from the partner’s sperm. The paternal pronucleus can be obtained in two ways: a. Fertilization of the donor oocyte with sperm and subsequent removal of the maternal pronucleus (46, 47) or b. Enucleation of a mature oocyte and subsequent fertilization by the partner’s sperm. In this case, only one pronucleus is formed. Polar body 2 can be transferred in the zygote with the male pronucleus and result in an embryo (48). The latter approach is the most appropriate in human, as it is hard to distinguish between male and female pronucleus (**Figure 1**).

NT techniques have been tested in several species, including non-human primates. Mice have been used as models in the vast majority of published papers to evaluate the efficacy of this technology due to their fast reproduction, easier manipulations, and abundancy of gamete cells. Another benefit of making use of these species is some conserved similarities with humans regarding the events of gametogenesis and early embryonic development (49). Following fertilization, the formation of the two pronuclei in the mammalian species signals the zygote formation. Zygote formation is followed by consecutive divisions of the newly formed embryo. Embryonic genome activation (EGA) is an important event that occurs after the first cell divisions, and it differs according to the species: in mouse: two-cell stage and in human between four and eight cells (50). Before EGA, the maternal proteins and mRNAS already present in the oocyte direct almost exclusively the first events of fertilization and embryonic development. Following EGA, embryonic cells compact and polarize to form two distinct cellular populations at the blastocyst stage. In mammals, embryos will implant at this stage, following the release of the blastocyst from the zona pellucida. Besides the similarities at these early embryonic stages, mice do display important differences when compared to larger mammals and humans. Embryonic developmental events occur faster in the mouse species. The events of EGA, cell division, compaction, blastulation, and implantation occur much faster in mice compared to human (49). Furthermore, mouse embryos have a prominent good embryonic development (reaching approximately 80% blastocyst rate) when cultured *in vitro*, when compared with larger mammalian species, including humans. In addition, meiotic errors in mouse oocytes are less evident than in human, with an estimated incidence of 1 and 5–20% respectively. Moreover, human embryos appear to have high numbers of mosaicism and aneuploidy rates than mouse embryos (49). Although mammalian species have served as good models to study the efficacy and safety of NT, species-specific differences usually demand a fine-tuning of the technology in the human species but also careful interpretation of the results.

For the abovementioned reasons, optimization of the technique in human oocytes was necessary before consideration of any future clinical application. These studies have focused on the efficiency of the NT technology using human oocytes regarding reconstruction and blastocyst rates and the amount of cytoplasmic carry-over from the oocyte that serves as a nuclear donor. A previous study by Craven et al. (51), performed PNT using human abnormally fertilized zygotes (1PN and 3PN). Reconstruction was successful, with less than 2% mitochondrial DNA carry-over from the nuclear donor. Nevertheless, a very low blastocyst formation rate was observed (8.3%) in comparison to unmanipulated controls (51). The PNT procedure was further optimized using normally fertilized zygotes and by refining the timing of the procedure. Early PNT, performed at 8 h post-ICSI was shown to be beneficial upon late PNT at 16 h post-ICSI. This adapted methodology was shown to significantly increase the blastocyst rates of the reconstructed zygotes, to a level of the non-manipulated controls (52). The first study to perform ST in human MII oocytes was published by Tachibana et al. (53), which confirmed the feasibility of the technique in human oocytes. Blastocyst rate in reconstructed oocytes following normal fertilization showed similar results with control fertilized oocytes (62 *vs* 76%). Human embryonic stem cells (hESCs) were also derived from the reconstructed embryos, carrying low levels of mtDNA from the nuclear donor (53). While ST and PNT have been mostly studied in the human, only one study has been reported making use of the PB1T strategy. Reconstructed PB1T oocytes were capable of normal fertilization, and PB1T zygotes developed to the blastocyst stage, but yet in a lower rate (42%) compared to the control group (75%) (54). PB2T studies in the human have been scarcely described, possibly due to the difficulty of distinguishing between male and female PNs (47). Mouse models using this technology have proven very promising but nevertheless, in the mouse it is easy to distinguish between female and male pronucleus, due to their evident size difference (47). The novel PB2T described by Tang et al. (48), optimized the use of PB2T in human oocytes and proved its efficacy. GVT remains challenging, as oocyte *in vitro* maturation is not yet optimized. In a mouse model, GVT oocytes were able to undergo maturation and cleave, but all embryos arrested before the blastocyst stage (55). In human, more studies are needed to improve the efficiency of the technique before it could be considered for clinical application, as still, *in vitro* maturated oocytes are inferior compared to their *in vivo* counterparts (56).

## Applications of Germline Nuclear Transfer

### Nuclear Transfer for Mitochondrial Diseases

NT techniques have initially been proposed to prevent the transmission of mtDNA diseases from the mother to the offspring. Mitochondrial disorders are reported to affect one in 5,000 individuals and are attributed to mutations or deletions in the mitochondrial DNA (mtDNA). Mitochondria are semi-autonomous organelles, important for the energy production and metabolism of the cell. They hold their own genome (mitochondrial DNA, mtDNA) in a variety of copies, coding for 37 genes. Other genes important for mitochondrial functions are encoded by the nuclear genome of the cell. Thus, mitochondrial function is under dual genetic control of both the mitochondrial and nuclear genome (57). Mitochondria are exclusively inherited only from the mother (58, 59) with a few rare exceptions being reported recently of possible paternal inheritance (60). Oocyte mtDNA mutations either reside in a homoplasmic state, with all of the copies carrying the mutation, or in a heteroplasmic state, presenting a mixture of mtDNA mutated and wild-type copies. The degree at which heteroplasmy occurs can differ between cells and tissues of one individual but can also shift in between generations. This can be attributed to the processes occurring during the formation of the female germline. At the stage of primordial germ cell formation, the number of mtDNA copies decreases significantly, which is a phenomenon designated as the mitochondrial genetic bottleneck (61). For women carrying heteroplasmic mtDNA mutations, this process can trigger a shift in heteroplasmy levels in the produced oocytes and makes it therefore difficult to predict the mutational load (number of affected mtDNA copies) in the corresponding generated embryo. Until now, there are no treatments available to eliminate mitochondrial diseases, only ways of prevention (62).

Pre-implantation genetic testing (PGT) has been used to determine the level of pathogenetic mtDNA copies in *in vitro* generated embryos in order to select mutation-free or mutation-low embryos which will not be affected by a mitochondrial disease (63). The mutational load should be less than 18% for an embryo to be considered safe for transfer, as calculated by a meta-analysis study by Hellebrekers et al., regardless the type of mtDNA mutation (64). Nevertheless, PGT might have diagnostic limitations, as embryonic mitochondria may shift their heteroplasmy levels during cell division, and mitochondrial mutations may be favored in response to environmental influences over wild type copies. In addition, patients carrying homoplasmic DNA mutations cannot be helped by PGT (65).

The NT technology can be beneficial for both patients carrying homoplasmic mutations, as well as for patients carrying heteroplasmic mutations for which no embryos with mtDNA mutational levels below 18% can be identified by PGT. By transferring the nuclear genetic material of the patient’s diseased oocyte/zygote to an enucleated, donated oocyte/zygote, containing healthy mtDNA copies, the reconstructed embryo is genetically related to both parents, with mtDNA being associated to the oocyte or zygote donor (66). During NT, it is inevitable that a minimal amount of cytoplasm is being transferred along, so a certain amount of the patient’s mtDNA copies will be present in the reconstructed NT embryos as well, which is known as the carry-over (67).

NT is a quite controversial topic in the field of ART, as it remains a new technology, and little is known about the effect on the health of the offspring. Studies in animal models demonstrated the feasibility of this technique and the potential to prevent mitochondrial diseases (47, 55, 68). A number of preclinical studies in human have also reported the carry-over of the patient’s oocyte to the donor’s cytoplasm and also verified the efficacy of PNT, ST, and PBT (52–54, 69). The first live birth in human was published in 2017, where the NT technology was used to overcome the transmission of Leigh syndrome, a mitochondrial disease, to the offspring (70). The patient had a long history of undiagnosed pregnancy losses and offspring death due to the disease. Following ST of the patient’s oocytes into enucleated oocytes of a donor and subsequent fertilization with the partner’s sperm, a healthy baby was born. The offspring was carrying low levels of mutant maternal mtDNA (2.36–9.23% in the tested tissues), indicating both the efficacy of the technique to prevent mitochondrial diseases, as well as the occurrence of carry-over (70). As the US is currently restricting the use of NT for infertility treatments, the patient was treated in Mexico. To date, one center in the UK is already applying NT technology to overcome the transmission of mtDNA diseases, and is extensively following up the health of the babies born (71, 72).

### Nuclear Transfer for Female-Related Infertility Treatments

#### Nuclear Transfer to Treat Fertilization Failure and Embryo Developmental Arrest

Infertility affects 8% to 12% of couples worldwide, and both female and male factors may contribute to it (73). The evolution of ART has helped many couples worldwide to deliver a healthy baby, but the treatment of some couples remains a challenge. Two not well characterized cases of infertility are failed fertilization (FF) following ICSI and embryo developmental arrest (EDA) (74, 75).

Although ICSI has offered promising results in the field of ART with fertilization rates of 70% to 80%, FF still occurs in 1% to 5% of ICSI cycles (76). Oocyte activation deficiencies are the main reason for FF and can be attributed to both oocyte- and sperm-related factors. Following sperm injection, the sperm initiates a series of events in order to activate the arrested metaphase II oocyte. Following fertilization, a rise in Ca^+2^ peaks occurs within the oocyte, which is important for its activation and subsequent embryo development (77). In patients with FF after ICSI, these peaks can be abnormal or absent. Currently, assisted oocyte activation (AOA) has been proven beneficial for most of these patients. AOA involves the production of Ca^+2^ oscillations artificially by different methods, such as the use of calcium ionophores (78, 79). Albeit promising for FF related to sperm-related deficiencies (80), when it comes to oocyte-related factors, AOA is not always efficient in these patients (81), who have to seek for oocyte donation (82). Oocyte factors are attributed to compromised cytoplasmic quality, such as reduced mitochondrial numbers or abnormal proteins involved in fertilization (83, 84). Up to now, only mutations in four female genes (*PATL2*, *WEE2*, *TLE6* and *TUBB8*) have been linked to FF (85), while AOA was not beneficial for women with *WEE2* mutations (86, 87). Nevertheless, injection of the *WEE2* cRNA led to successful activation of the affected oocytes, allowing the formation of blastocysts (85), suggesting that cytoplasmic incompetence can be overcome by enriching the oocyte with the normal cRNA. The use of NT could possibly help these patients when a sperm-related factor is excluded. The compromised cytoplasm of the affected oocytes could be replaced by the cytoplasm of a donor oocyte by transferring the genetic material of the patient to the donor oocyte. There are currently no publications suggesting the use of NT to rescue FF in patients with oocyte activation deficiency factors, as oocyte factors are not yet well characterized. Oocyte factors are complex to study, not only because oocytes are scarce for research purposes, but also because a large number of maternal factors are involved in the oocyte activation process (88). Specifically, errors can occur in the oocyte Ca^2+^ realizing machinery, in the pathways activated downstream the Ca^2+^, in the channels and pumps involved in Ca^2+^ homeostasis, but also due to a poor overall oocyte quality or nuclear defects (88).

Another not well understood condition is embryo developmental arrest. EDA is characterized by the primary arrest of embryos in the early cleavage stages (75). Approximately, 10 to 15% of IVF embryos arrest permanently, and some patients present recurrent complete embryo developmental arrest (89). Before embryonic genome activation (between four and eight cells in human), embryonic development is almost exclusively regulated by maternal RNAs and proteins stored in the cytoplasm (50). The genes encoding for these essential maternal factors are the so-called maternal effect genes (MEGs). Over 60 oocyte-specific MEGs have been found to be critical for mammalian development (90). However, research in human is still limited, and only few MEGs, some of which form the subcortical maternal complex (SCMC) have been identified. The SCMC is a multiprotein complex, composed of at least six proteins, that participates in the zygote genome activation, but its exact functions are still under debate (91). Recently, mutations in the genes involved in the formation of the SCMC, such as *TLE6*, *PADI6*, *NRLP2*, *NRLP5*, *NRLP7*, and *KHDC3L* have been detected in patients suffering from EDA (92–96). For patients facing EDA, the only current solution remains oocyte donation. As a treatment to embryo developmental arrest, NT could be proposed.

Two recent publications (42, 43) investigated the use of NT technology to overcome embryo developmental arrest in a mouse model. Nuclear transfer (both ST and PNT) between oocytes from NZB/OlaHsd mice that display a two-cell blockage and control B6D2 mice, rescued the embryonic development, resulting in high blastocyst rates (42). The use of ST by of Costas-Borges et al (43), using the same mouse model, demonstrated similar results but also low carry-over rates of maternal mitochondrial DNA and low heteroplasmy levels in the offspring for several generations, as well as normal fertility of the pups from the reconstructed embryos (43).

A recent study by Bai et al. (97), reported on the use of different NT techniques to overcome embryo developmental arrest in a *Zar1*−/− mouse model. *Zar1* is an important regulator of maternal genome degradation and embryonic genome activation. *Zar1*−/− mice displayed embryo developmental arrest. In order to rescue the development of these embryos, NT technology was applied, including ST or PB1T between *Zar1*−/− and wild type mouse oocytes, or early and late PNT between *Zar1*−/− and wild type zygotes. ST, early PNT, and PB1T significantly increased the blastocyst stage of the reconstructed oocytes/zygotes and also led to live offspring in 17.2% for early PNT, 32.6% in the ST group, and 29% for the PB1T group, comparable to the control group. Furthermore, the resulted offspring were healthy and fertile. Nevertheless, the delivery rate for late PNT was only 2.82% in the reconstructed zygotes (97).

In human, the first NT report to overcome embryonic arrest was reported in 2016 by Zhang et al. (98). PNT was applied for a patient with recurrent embryonic arrest at two-cell stage, following fertilization. The transfer of the pronuclei into the zygote of a donor, resulted in five four-cell stage embryos and a triplet pregnancy, although no live birth was achieved (98).

FF and EDA remain the challenges for ART, and cytoplasmic oocyte quality is of great importance for appropriate embryonic development. Current data are encouraging but not sufficient to support the use of NT for infertility treatment. Yet, two clinics in Greece and Ukraine are claiming live births by making use of the NT technology for female-related infertility, but peer-reviewed publications are currently lacking.

#### Nuclear Transfer for Advanced Maternal Age

In IVF clinics, women over the age of 37 years remain a challenging population for ART. Advanced maternal age is accompanied by ovarian aging, which is characterized by a decline in both quantity and quality of oocytes (99). Poor oocyte quality in aged women is associated with cytoplasmic deficiencies and impaired mitochondrial function, which has a negative impact on the ATP supply to support oocyte maturation and embryo development (100, 101). The mtDNA copies in a cell are directly correlated with its metabolic needs. In mature human oocytes, for instance, the number of mtDNA copies is approximately 100,000–600,000 (62). Importantly, a threshold of mtDNA copies has been suggested for successful fertilization and subsequent embryonic development (101). Women of advanced maternal age generate more aneuploid embryos compared with younger women (102). An oocyte has high energy demands for the formation of the meiotic spindle and the correct alignment of the chromosomes, but also to complete maturation, fertilization, and support the first cleavage stages of embryonic development (101). Mitochondria are maternally inherited, and no mitochondrial replication occurs before the blastocyst stage. Thus, the number of mtDNA copies in the oocyte is important for the first steps of embryonic development (100). The mtDNA copy number in the oocytes of older women is significantly decreased compared to those of younger women (103). This number is also reduced in the early cleaved embryos, while it is higher in blastocysts of older women. Nevertheless, this high number of mtDNA copies in blastocysts of older women has been associated with increased aneuploidy and failed implantation (104).

Despite the mitochondria, other cytoplasmic factors are also important for fertilization and proper embryonic development, such as organelles, metabolites, maternal RNAs and proteins, as descripted in the previous section (38, 105). A recent study by Bertoldo et al. (106), reported that poor oocyte quality from reproductive aged mice was associated with reduced levels of the metabolic cofactor nicotinamide adenine dinucleotide (NAD^+^). Supplementation of the NAD^+^ precursor Nicotinamide mononucleotide restored oocyte quality and enhanced blastocyst quality and live birth rates in the aged females (106).

Several methodologies have been explored to overcome the poor cytoplasmic quality of women with advanced maternal age. Cytoplasm transfer (CT), which involves injection with a limited portion of cytoplasm from a competent (donor oocyte) to an incompetent oocyte (107) and Autologous Germline Mitochondrial Energy Transfer (AUGMENT) have been investigated (108). The safety and benefit of CT remain unclear owing to certain abnormalities observed in the resultant children (109) although it is not certain that these abnormalities were caused by CT. Clinical applications of this technology were put into practice before extended animal studies, although a recent paper from Tang et al. demonstrated that CT was not beneficial to overcome cytoplasmic inferiority of the oocytes from old mice, in contrast to NT (42). Alternatively, the method of AUGMENT has been investigated. AUGMENT involves the supplementation of the incompetent oocyte with autologous mitochondria, isolated from oogonial stem cells harvested from an ovarian biopsy of the patient (108). Nevertheless, it is difficult to confirm the efficacy of the technique due to the small number of patients treated and also due to the difficulty of isolating oogonial stem cells and the controversy around their existence in the human adult ovary (110–113). Furthermore, a recent study reported no benefit in embryo quality in women with multiple IVF failures using the AUGMENT technology (111).

NT has been proposed for the indication of advanced maternal age. In 2009 Mitsui et al. (114), demonstrated the effectiveness of ST to rescue poor development in embryos originating from aged mice. Oocytes from young mice were used as recipients and high blastocyst rates were achieved (114). Tang et al. (42) achieved also promising results making use of ST and PNT. Furthermore, spindle assembly and mitochondrial potential were assessed in oocytes of mice of advanced age. A significantly higher number of abnormal spindles and misaligned chromosomes were noticed in the oocytes of aged and very aged mice compared with oocytes from young mice (42). Furthermore, mitochondrial membrane potential, representative of mitochondrial function, was severely compromised in the aged and very aged mouse group. Mitochondrial membrane potential values were increased in reconstructed oocytes with spindles from very aged mice transferred in the cytoplasm of young mice (42). Fertilization and blastocysts levels following sperm injections were significantly lower in the aged and very aged mouse group compared to the one of young mice. PNT significantly increased the fertilization and blastocyst rate of the reconstructed oocytes in both aged and very aged groups, after transfer of the pronuclei into enucleated zygotes from young oocytes. ST also increased fertilization and blastocyst formation for the reconstructed oocytes of the aged group but did not improve the results in the very aged mouse oocytes. Importantly, euploidy rate was very high in embryos originating from the reconstructed NT oocytes/zygotes. Opposite results were observed with the transfer of the spindle or pronuclei from young mice in the cytoplasm of very aged mice (42). These results could indicate that ST and PNT might be able to avoid embryo aneuploidies created during embryo development, caused by the poor cytoplasmic quality of oocytes from mice of advanced maternal age. Nevertheless, the number of blastocysts analyzed in this paper was limited, and the results should be translated cautiously.

#### Use of Nuclear Transfer Technology for DOR/POR Patients

DOR patients are associated with poor reproductive outcomes, even when ART techniques are used (14). DOR patients usually exhibit POR due to compromised ovarian reserve (115). Poor oocyte numbers following stimulation regardless of the age of the patient or embryo quality have been associated with poor clinical results in this patient group (16). POR patients appear to be in a higher risk for foetal aneuploidies compared to normal response and higher chances for pregnancy loss, Down syndrome and other embryonic aneuploidies have been associated with patients with DOR (116–118).

In aged women with POR, poor pregnancy rates have been reported, as a normal sequence of ovarian ageing. As previously described, advanced maternal age is characterized by poor oocyte cytoplasm which highly compromises embryonic development and pregnancy rates (119). Despite the normal fertility decline associated with age, it is unclear whether this patient group is associated with higher embryonic aneuploidies compared with age matched control women. POR patients have similar fertilization, implantation, aneuploidy, and miscarriage rates compared to aged women with normal response to gonadotropins (16). Nevertheless, the number of embryos available significantly decreases in POR patients, affecting the chances for embryo transfer (16).

Poor oocyte and embryo quality do not seem to be the case for young women with POR (37, 39, 40). Young patients with POR seem to have similar fertilization rates and good embryo quality compared to age matched control women. Nevertheless, again, the number of oocytes retrieved following stimulation affects the number of available embryos for embryo transfer, resulting in decreased implantation and LBR. When a blastocyst is acquired, LBR is comparable to the women with normal ovarian reserve (39). One of the most important factors in the outcome of ART is the number of recruited oocytes following ovarian stimulation (120). A good yield of oocytes renders higher chances for a sufficient number of euploid embryos (121).

**Figure 2:**
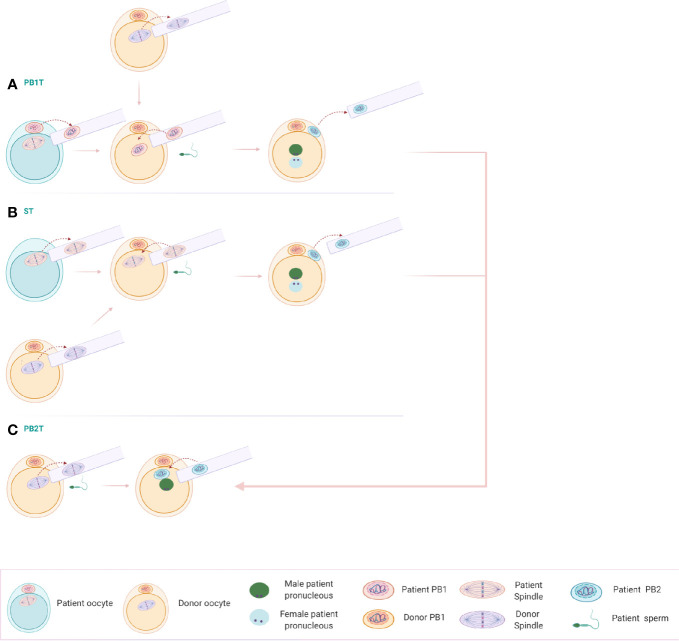
**(A)** Polar body 1 transfer (PB1T): The first polar body of a mature oocyte is transferred to a donor mature oocyte from which the spindle has been removed. Following reconstruction, the oocyte is fertilized, extruding the second polar body. **(B)** Spindle transfer (ST): The spindle of the patient’s oocyte can be transferred into an enucleated donor metaphase II (MII) oocyte. The reconstructed oocyte can be fertilized with the patient’s sperm and extrude the second polar body. **(C)** Polar body 2 transfer (PB2T): An oocyte of the donor is enucleated and fertilized with the sperm of the patient’s partner. A single pronucleus is being formed, containing only the genetic material of the partner. Polar body 2 resulting from PB1T or ST can be transferred to the zygote, including now the genetic material of the patient and the correct genetic load.

Since cytoplasmic quality and oocyte numbers are important factors for the outcome in the ART setting, NT could assist these patients. For the patients with advanced maternal age, DNA from the patient’s oocytes could be transferred to the cytoplasm of a healthy young donor. In addition, the low number of oocyte yields from DOR/POR patients could be overcome by the use of NT. Here, we are proposing the use of three different NT techniques that would yield four embryos instead of one, starting from one patient oocyte (**Figure 2**).

The first step involves the transfer of the 1^st^ polar body (PB1T) into an enucleated mature oocyte of a donor. Following fertilization with the sperm of the patient’s partner, second polar body extrusion occurs with the formation of pronuclei, belonging to the patient’s genomic DNA and the one of her partner’s. The remaining spindle in the patient’s oocyte can be transferred to a second enucleated donor oocyte and results in a zygote and second polar body extrusion following fertilization. Two more donor oocytes can be used for the reconstruction of embryos using the two second polar bodies after ST and PB1T. Donor oocytes are enucleated and fertilized with sperm from the partner of the patient. A single male pronucleus is formed. The second polar bodies from the reconstructed oocytes can be transferred to the zygote and results in two more embryos. The technique of this novel PB2T in human has been successfully optimized recently by Tang et al. in a research setting (48).

According to the above scheme, making use of only one patient oocyte, four embryos could be reconstructed with the help of NT technology. This would allow more available embryos to be chosen for embryo transfer in this patient group.

## Challenges in Nuclear Transfer Technology

Despite the promising results of the NT technology in some animal and human models, results remain scarce on the value of this technique in clinical practice when mitochondrial diseases are not involved. Furthermore, a number of concerns have been raised over the years about the safety of the technique for the offspring.

Since mitochondrial function is under dual control of both nuclear and mtDNA, concerns are raised towards the possible incompatibility of the nuclear DNA (patient) and mtDNA (donor), which may occur from different mtDNA haplogroups. Diversity in the mtDNA sequence has been identified between individuals, with different haplotypes in mtDNA copies. Individuals fall in different haplogroups, depending on the characteristics of the mtDNA variants. These haplogroups were established during human evolution, and they are characteristic of some geographical regions (66). Nuclear–mitochondrial incompatibility has been previously reported in an animal study, reporting high embryonic lethality and stillborn rates in mice, when applying nuclear transfer between two mouse breeds (122). Nevertheless, health reports in other studies suggest that nuclear–mitochondrial incompatibility is not an issue (68, 70). Furthermore, current evidence also suggests that nuclear transfer does not affect the mitochondrial function in humans. This was shown in Embryonic stem cells (ESCs) from reconstructed blastocysts using NT. ESCs had similar mitochondrial respiratory chain enzyme activity and oxygen consumption rates regardless the combination of nuclear–mitochondrial DNA (53, 123).

Another reason for caution when applying NT is the occurrence of cytoplasmic carry-over of mutant mtDNA molecules from the patient’s to the donor oocyte. Even though reported levels of mtDNA carry-over in reconstructed NT embryos were always lower than the 18% threshold level for disease expression (51, 52), the occurrence of heteroplasmy drift could cause a shift in heteroplasmy levels during development. A recent study demonstrated a competition between different mtDNA haplotypes. The heteroplasmic mouse model containing the C57BL/6JOlaHsd nuclear genome and either NZB/OlaHsd or C57BL/6JOlaHsd mtDNA showed that one of the mtDNA haplotypes was becoming predominant, termed as “haplotype selection”, during oogenesis and early embryo development, which was dependent on the specific interaction between the nuclear and mitochondria encoded genes (61). Despite the low heteroplasmy levels in reconstructed human embryos, progressive increase in the mtDNA heteroplasmy levels of several hESC lines derived from reconstructed NT blastocysts has been reported (52, 123). Whether this is due to the artificial nature of hESCs or a biological phenomenon has to be further explored. Since the effect of heteroplasmy on the reconstructed embryo has not been elucidated yet, the minimal carry-over should be guaranteed. The most studied nuclear transfer technologies are ST and PNT. Several studies have reported different levels of carry-over to the reconstructed embryos by the application of the two techniques; nevertheless it seems that these two techniques allow a very small amount of mtDNA to be transferred from the nuclear donor. The minimum carry over seems to occur with the use of PBT, as polar bodies have a very small cytoplasmic content (47, 62).

Another limitation for the use of NT technology is that it would increase the financial cost for the patients. This should be avoided until the benefits and the safety of the technique are more concrete for patients with female subfertility. Nevertheless, these techniques are not so labor intensive, as especially the model we are proposing can be done during the daily IVF routine, and PB2T can occur early the next morning. Yet, a microscope for spindle visualization is required and due to the sensitivity of the material, users should be well-trained. It is worthy to note that NT cannot correct all aspects of infertility. If genetic anomalies already exist in the spindle or polar bodies of the mother, something that is highly prevalent in aged women (124), then the NT will not be of benefit (42). In this regard, PGT to assess aneuploidy should be offered in all cases when NT embryos are being created as the NT technique is still very novel.

Before any further application of this technology, the ethical aspects should be considered thoroughly.

The ethical concerns primarily refer to the genetic modification of the germline. Some argue that NT could cause genetic modifications that could be transmitted to the next generation. Nevertheless, unlike other technologies, NT does not target the DNA of the genome nor of the mitochondria (125). Although strict regulations and federal organizations have been established in some countries that control the creation of embryos for research purposes, prohibiting any use for eugenic intent, the use of human embryos remains a debatable issue (126). Furthermore, a number of people argue that women donating oocytes for research could be exploited, and the donated oocytes could be seen as a commodity to experiment the different research techniques (127). The genetic contribution of the donors has also been criticized. Nevertheless, in the case of mitochondrial donation, nuclear DNA from the donor is not contributing to the offspring, in contrast to other cases of oocyte donation, only the mitochondrial DNA. Finally, one of the most important ethical concerns for the use of NT is the safety of this technique to the offspring (127). Although available studies are promising, the number of applications in human is limited, and therefore, the technique should be considered as highly experimental, and thorough follow-up of the children born after this technology should occur.

## Conclusions

DOR patients remain a challenge for current ART practice. Poor response to gonadotropins and poor oocyte quality lead to the recruitment of a lower number of good quality oocytes for fertilization. NT is a new technology being used to overcome the transmission of severe mitochondrial diseases from mother to offspring (128). Lately, this technology has been proposed for certain types of female-related subfertility (98), but scientific reports remain scarce. Making use of the proposed NT scheme described above, we believe that a higher number of embryos can be reconstituted for DOR patients when making use of these various NT strategies. This approach is expected to also overcome cytoplasmic defects in oocytes of women of advanced age. Before any application of NT for DOR patients, more studies should be carried out in animal models before assessing the safety of the technique on patients suffering from subfertility and PGT should remain the tool to ensure the safety of the reconstructed embryos.

## Author Contributions

AC and BH designed the idea of the review. AC and BH wrote the manuscript. AB, MT, CD, and DS provided scientific input, corrected, and edited the manuscript. All authors contributed to the article and approved the submitted version.

## Funding

AC is a holder of an FWO funding: FWO-Vlaanderen (Flemish AC is a holder of an FWO funding: FWO-Vlaanderen (Flemish Fund for Scientific Research,1S80220N). BH has been awarded with a BOF (Bijzonder Onderzoeksfonds) GOA (Geconcerteerde onderzoeksacties) 2018000504 (GOA030-18 BOF) funding and FWO-Vlaanderen (Flemish Fund for Scientific Research, G051516N and G1507816N). China Scholarship Council (CSC) (201506160059), FWO-Vlaanderen (Flemish fund for scientific research, Grant no. G051017N) and Special Research Fund from Ghent University (Bijzonder Onderzoeksfonds, BOF) (01SC2916 and 01SC9518) have been awarded to MT.

## Conflict of Interest

The authors declare that the research was conducted in the absence of any commercial or financial relationships that could be construed as a potential conflict of interest.
